# Supplementary figures and images for: Underrepresentation of women in computer systems research
Source: PLoS One. 2022 Apr 6;17(4):e0266439. doi: 10.1371/journal.pone.0266439 (PMC8985950; doi:10.1371/journal.pone.0266439)

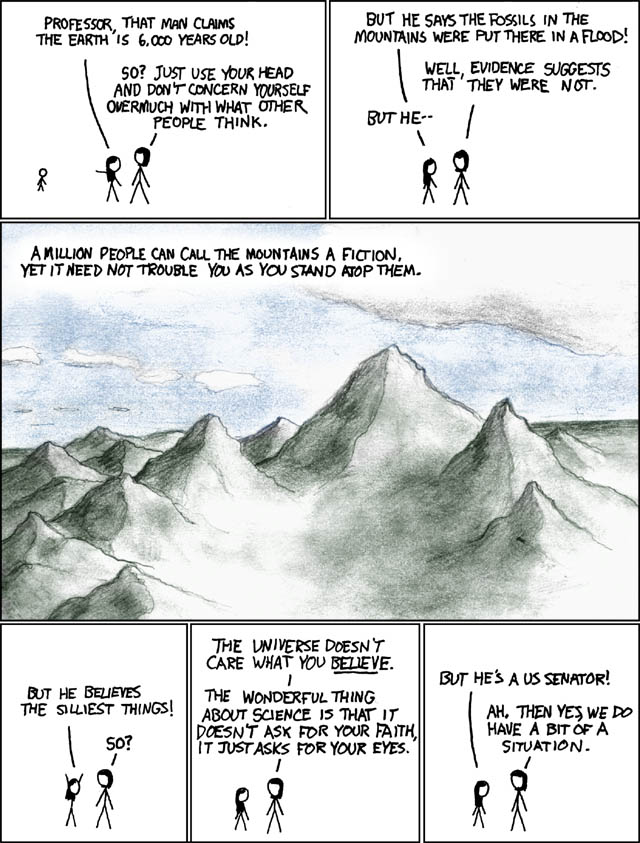

Supplement: S1 Dataset — (ZIP) [file pone.0266439.s001.zip › sysconf/docs/images/beliefs.jpg]

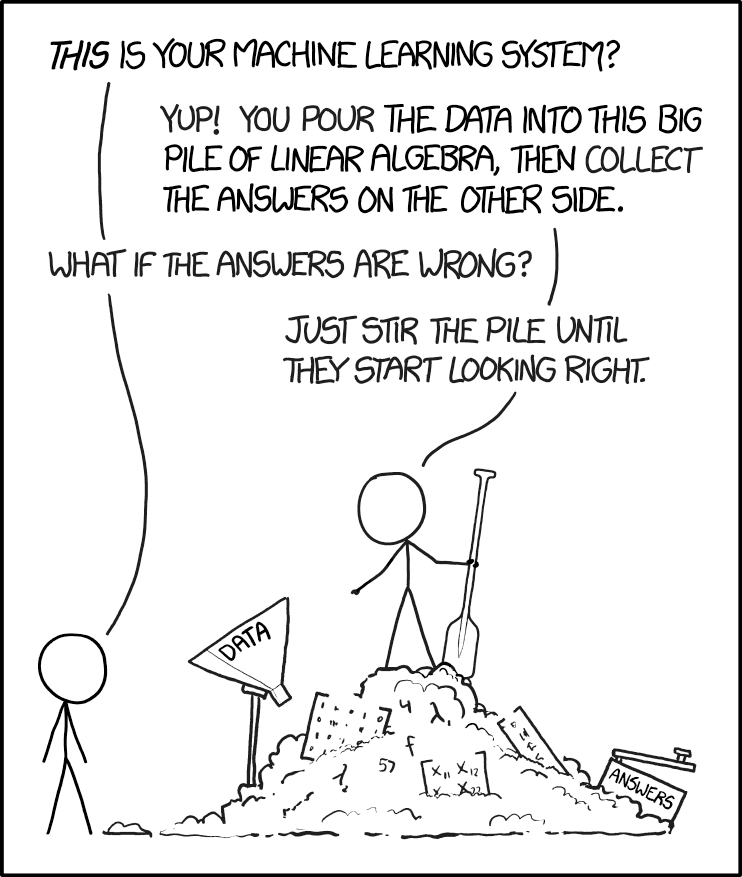

Supplement: S1 Dataset — (ZIP) [file pone.0266439.s001.zip › sysconf/docs/images/machine_learning_2x.png]

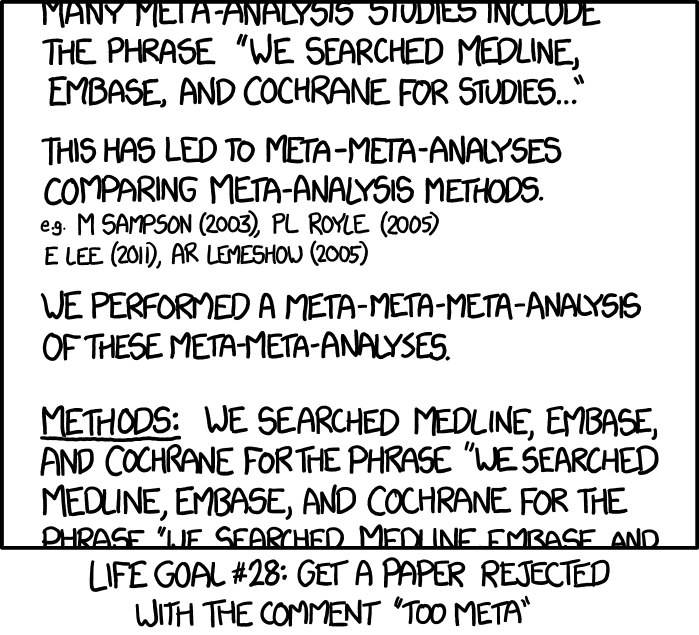

Supplement: S1 Dataset — (ZIP) [file pone.0266439.s001.zip › sysconf/pubs/web/images/meta-analysis_2x.png]

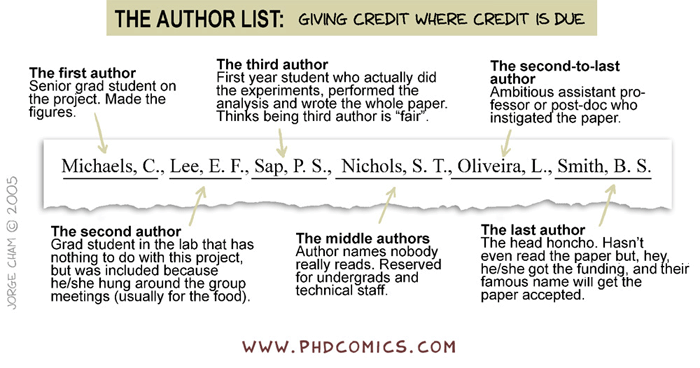

Supplement: S1 Dataset — (ZIP) [file pone.0266439.s001.zip › sysconf/pubs/web/images/phd031305s.gif]

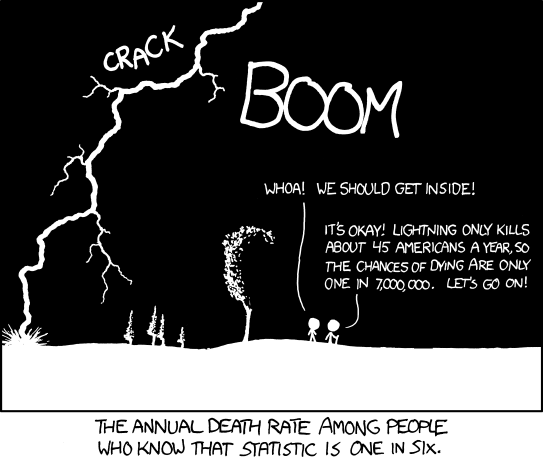

Supplement: S1 Dataset — (ZIP) [file pone.0266439.s001.zip › sysconf/pubs/web/images/conditional_risk.png]

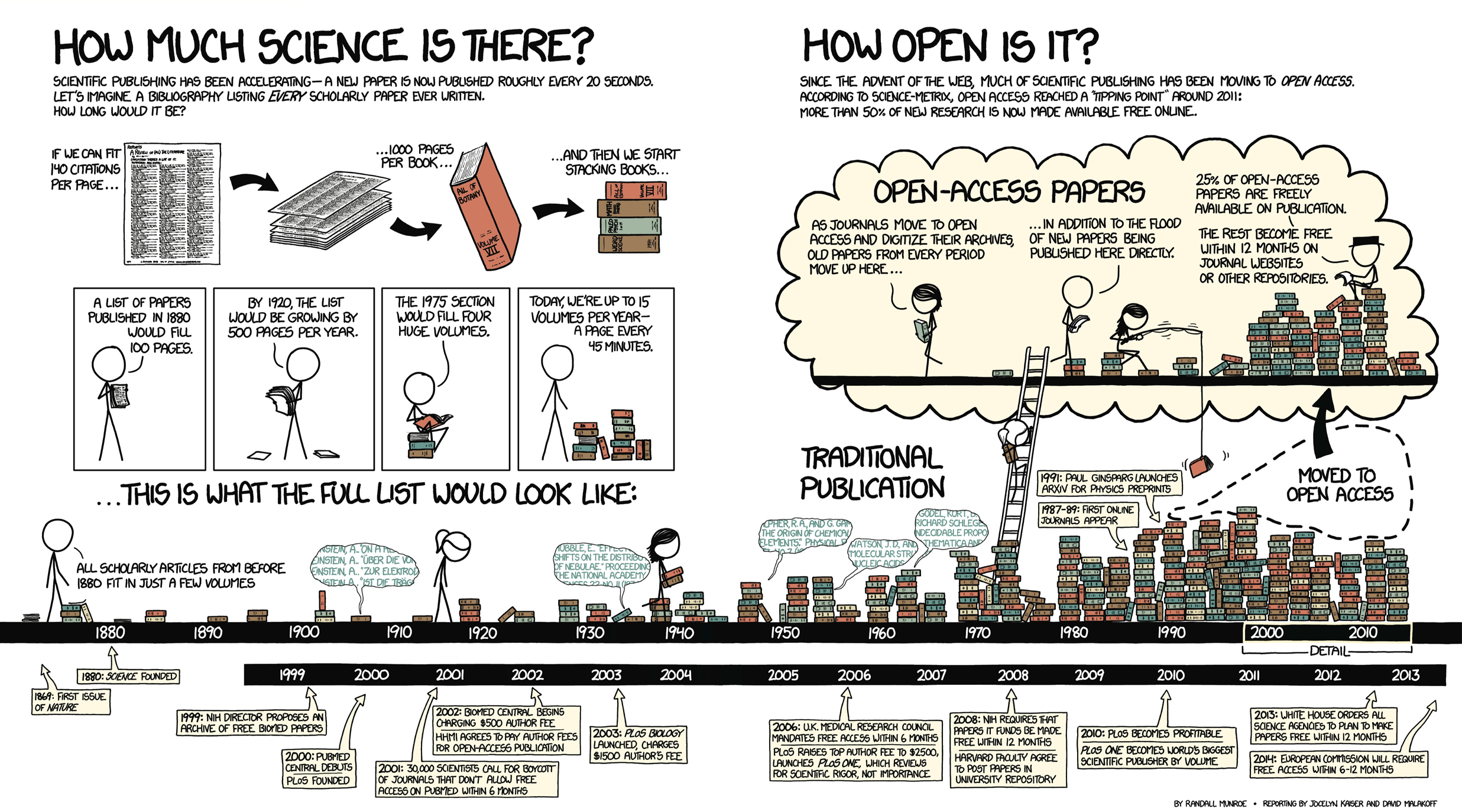

Supplement: S1 Dataset — (ZIP) [file pone.0266439.s001.zip › sysconf/pubs/web/images/infographic.jpg]

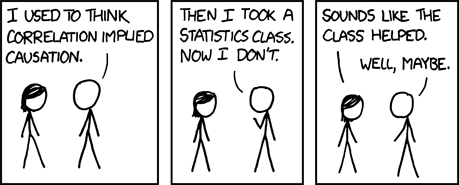

Supplement: S1 Dataset — (ZIP) [file pone.0266439.s001.zip › sysconf/pubs/web/images/correlation.png]

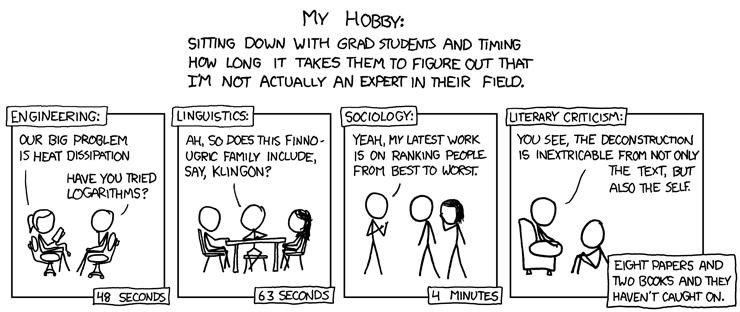

Supplement: S1 Dataset — (ZIP) [file pone.0266439.s001.zip › sysconf/pubs/web/images/impostor.png]

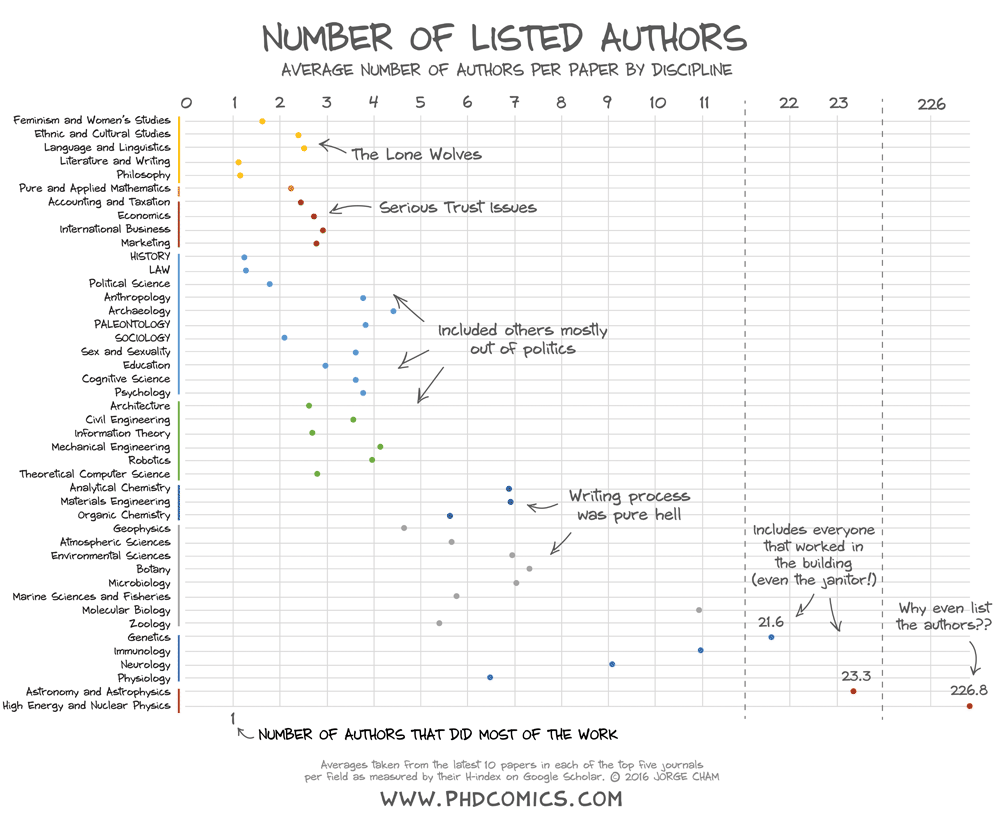

Supplement: S1 Dataset — (ZIP) [file pone.0266439.s001.zip › sysconf/pubs/web/images/phd120916s.gif]

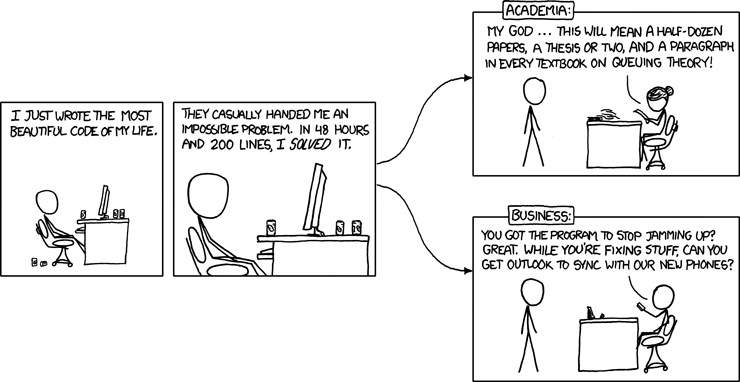

Supplement: S1 Dataset — (ZIP) [file pone.0266439.s001.zip › sysconf/pubs/web/images/academia_vs_business.png]

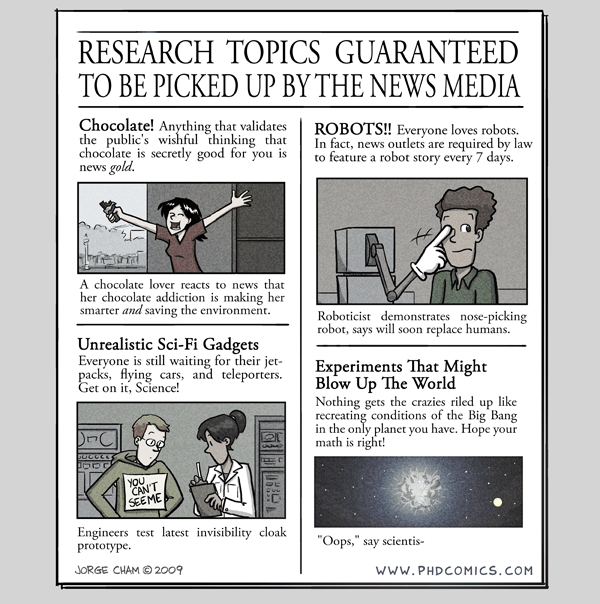

Supplement: S1 Dataset — (ZIP) [file pone.0266439.s001.zip › sysconf/pubs/web/images/phd052009s.gif]

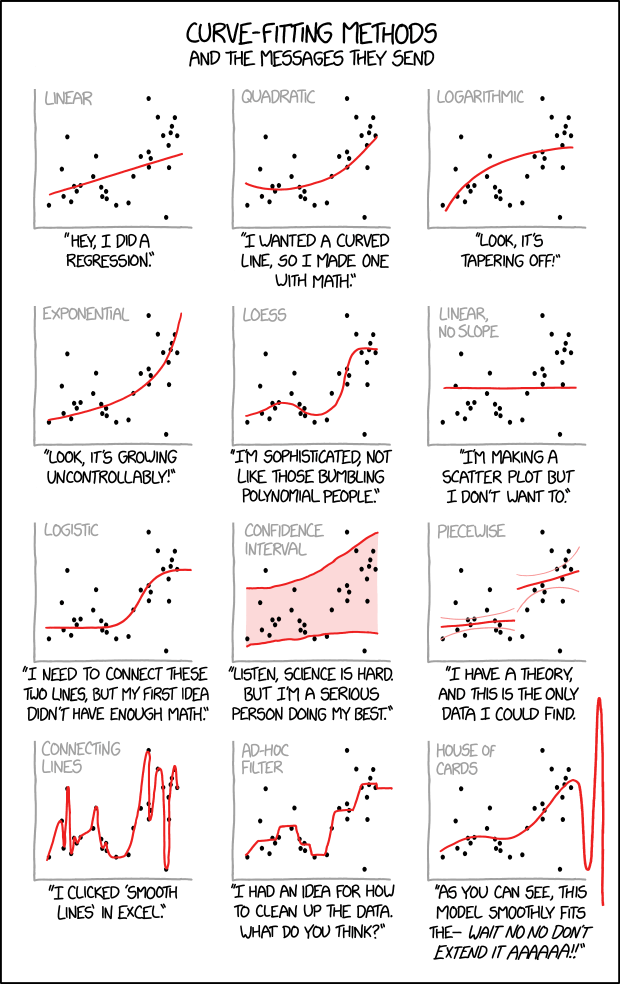

Supplement: S1 Dataset — (ZIP) [file pone.0266439.s001.zip › sysconf/pubs/web/images/curve_fitting.png]

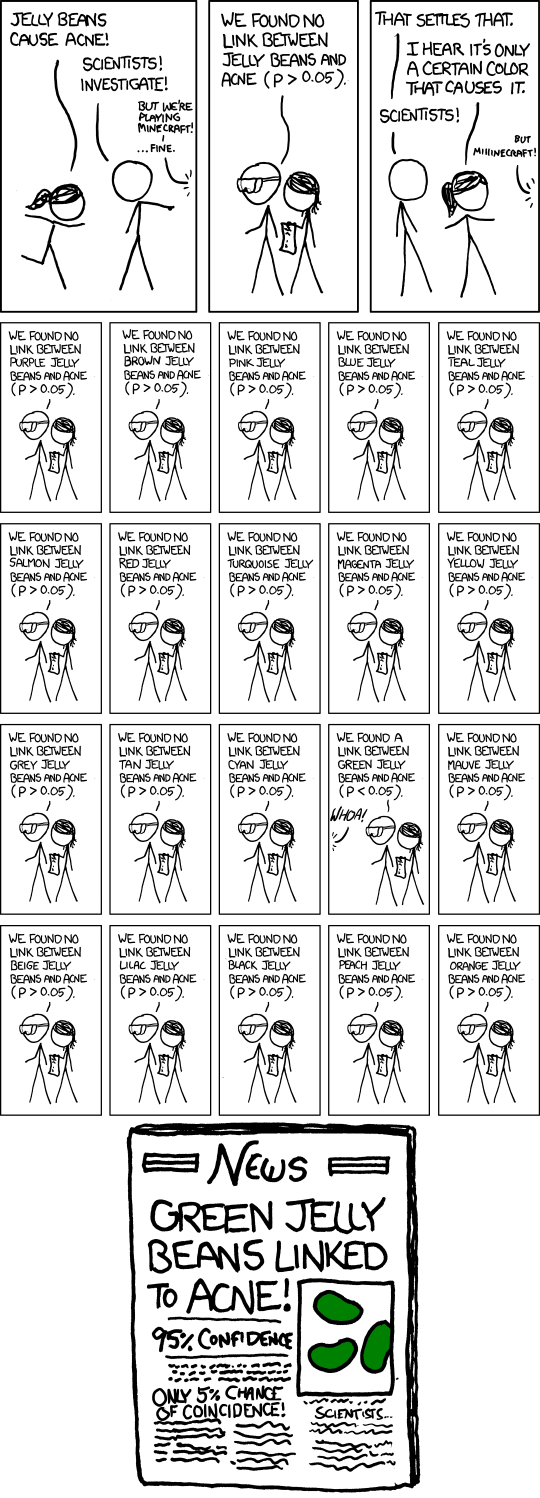

Supplement: S1 Dataset — (ZIP) [file pone.0266439.s001.zip › sysconf/pubs/web/images/significant.png]

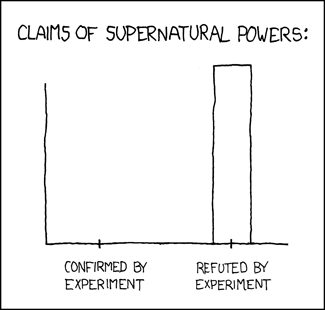

Supplement: S1 Dataset — (ZIP) [file pone.0266439.s001.zip › sysconf/pubs/web/images/the_data_so_far.png]

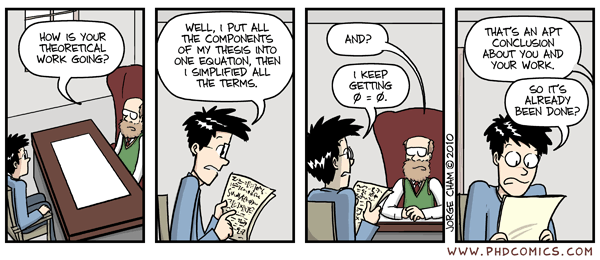

Supplement: S1 Dataset — (ZIP) [file pone.0266439.s001.zip › sysconf/pubs/web/images/phd110110s.gif]

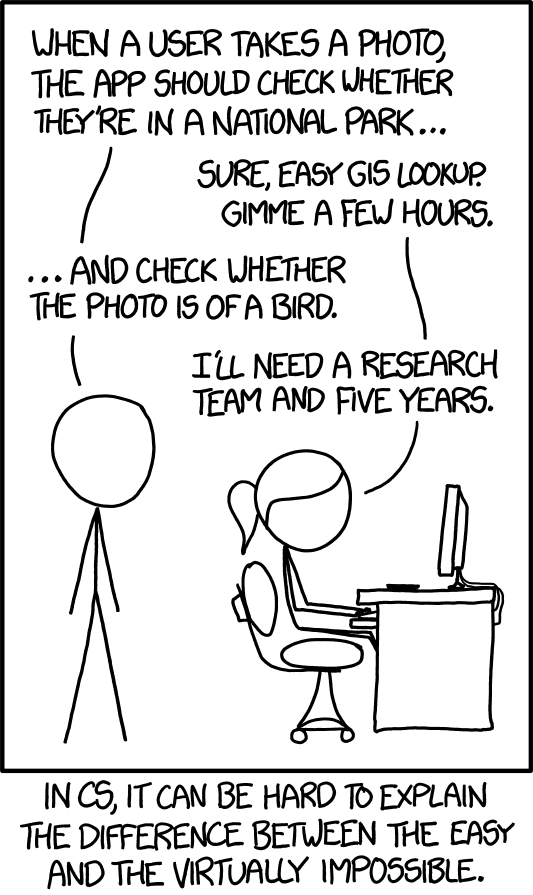

Supplement: S1 Dataset — (ZIP) [file pone.0266439.s001.zip › sysconf/pubs/web/images/tasks_2x.png]

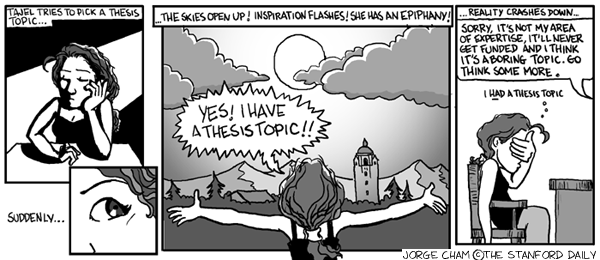

Supplement: S1 Dataset — (ZIP) [file pone.0266439.s001.zip › sysconf/pubs/web/images/phd100998s.gif]

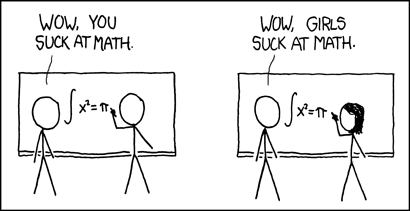

Supplement: S1 Dataset — (ZIP) [file pone.0266439.s001.zip › sysconf/pubs/web/images/how_it_works.png]

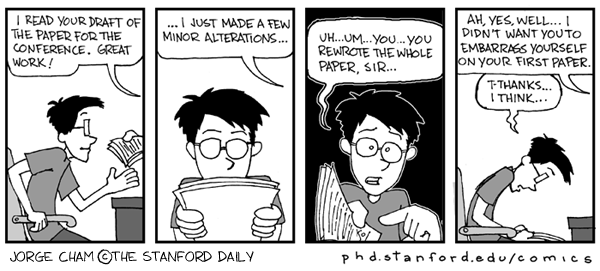

Supplement: S1 Dataset — (ZIP) [file pone.0266439.s001.zip › sysconf/pubs/web/images/phd010500s.gif]

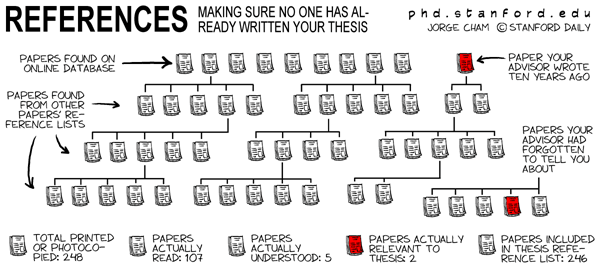

Supplement: S1 Dataset — (ZIP) [file pone.0266439.s001.zip › sysconf/pubs/web/images/phd022702s.gif]

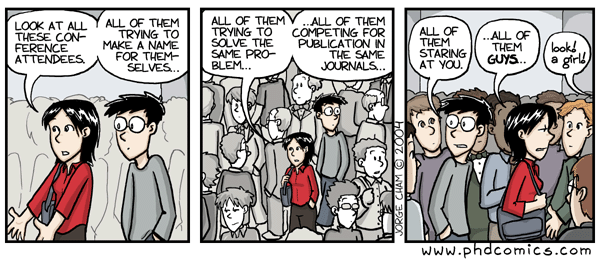

Supplement: S1 Dataset — (ZIP) [file pone.0266439.s001.zip › sysconf/pubs/web/images/phd081604s.gif]

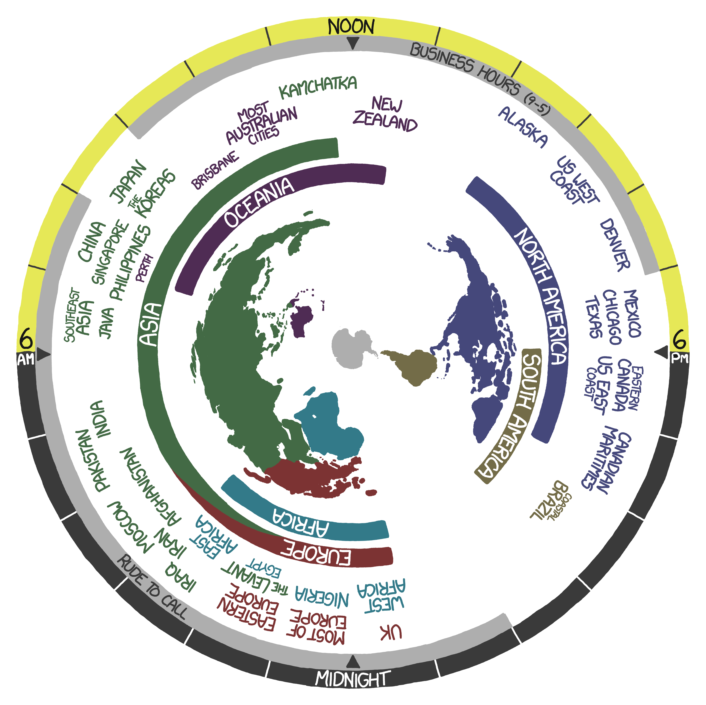

Supplement: S1 Dataset — (ZIP) [file pone.0266439.s001.zip › sysconf/pubs/web/images/11h30m.png]

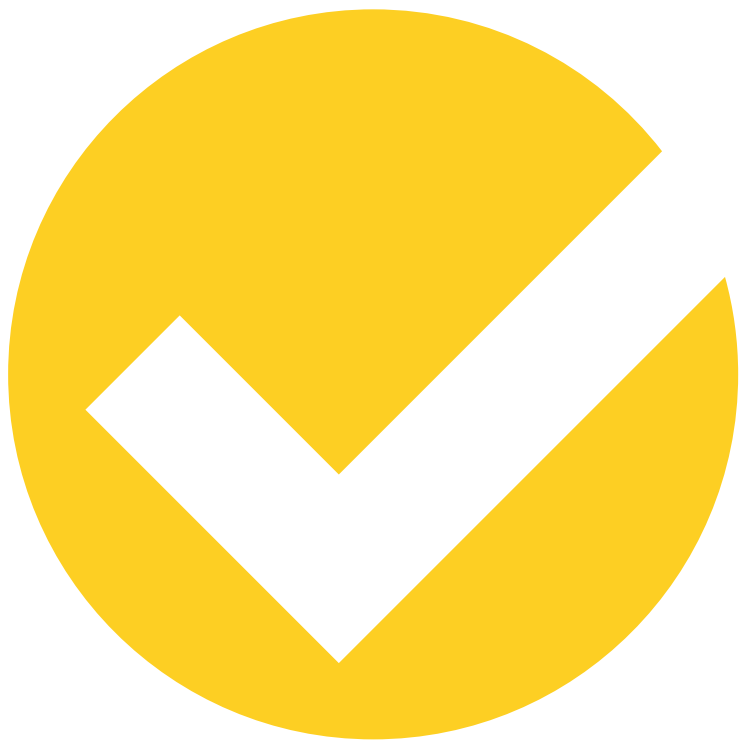

check for  
updates

Supplement: S1 Dataset — (ZIP) [file pone.0266439.s001.zip › sysconf/pubs/collab/Definitions/logo-updates.pdf]

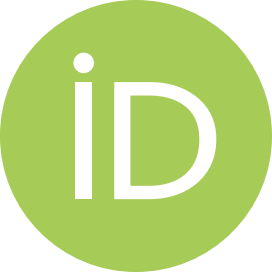

Supplement: S1 Dataset — (ZIP) [file pone.0266439.s001.zip › sysconf/pubs/collab/Definitions/logo-orcid-eps-converted-to.pdf]
