# Supplementary material for: Underrepresentation of women in computer systems research
Source: PLoS One. 2022 Apr 6;17(4):e0266439. doi: 10.1371/journal.pone.0266439 (PMC8985950; doi:10.1371/journal.pone.0266439)
Supplement: S1 Appendix — (PDF) [file pone.0266439.s002.pdf]

S2 Appendix. Detailed conference list

1. ASPLOS'17: ACM International Conference on Architectural Support for Programming Languages and Operating Systems, China, 2017-04-08. 320 submissions, 56 papers accepted (double-blind reviews). 247 total authors and 49 total PC members. homepage: `http://novel.ict.ac.cn/ASPLOS2017/`

2. ATC'17: Annual Technical Conference, United States, 2017-07-12. 277 submissions, 60 papers accepted (single-blind reviews). 279 total authors and 33 total PC members. homepage: `https://www.usenix.org/conference/atc17`

3. CCGrid'17: IEEE/ACM CCGrid, Spain, 2017-05-14. 286 submissions, 72 papers accepted (single-blind reviews). 296 total authors and 221 total PC members. homepage: `https://www.arcos.inf.uc3m.es/wp/ccgrid2017/`

4. CCS'17: Conference on Computer and Communications Security, United States, 2017-10-31. 836 submissions, 151 papers accepted (double-blind reviews). 589 total authors and 145 total PC members. homepage: `https://www.sigsac.org/ccs/CCS2017/`

5. CIDR'17: The biennial Conference on Innovative Data Systems Research, United States, 2017-01-08. 78 submissions, 32 papers accepted (single-blind reviews). 213 total authors and 30 total PC members. homepage: `http://cidrdb.org/cidr2017/`

6. CLOUD'17: International Conference on Cloud Computing, United States, 2017-06-25. 110 submissions, 29 papers accepted (single-blind reviews). 110 total authors and 129 total PC members. homepage: `http://www.thecloudcomputing.org/2017/`

7. Cluster'17: Cluster, United States, 2017-09-05. 217 submissions, 65 papers accepted (single-blind reviews). 273 total authors and 133 total PC members. homepage: `https://cluster17.github.io/`

8. CoNEXT'17: International Conference on Emerging Networking Experiments and Technologies, South Korea, 2017-12-13. 171 submissions, 32 papers accepted (single-blind reviews). 145 total authors and 48 total PC members. homepage: `http://conferences2.sigcomm.org/co-next/2017/#!/home`

9. EuroPar'17: International European Conference on Parallel and Distributed Computing, Spain, 2017-08-30. 176 submissions, 50 papers accepted (single-blind reviews). 179 total authors and 86 total PC members. homepage: `http://europar2017.usc.es/`

10. EuroSys'17: The European Conference on Computer Systems, Serbia, 2017-04-23. 188 submissions, 41 papers accepted (double-blind reviews). 169 total authors and 47 total PC members. homepage: `https://eurosys2017.github.io/`

11. FAST'17: USENIX Conference on File and Storage Technologies, United States, 2017-02-27. 116 submissions, 27 papers accepted (double-blind reviews). 119 total authors and 34 total PC members. homepage: `https://www.usenix.org/conference/fast17/`

12. HCW'17: International Heterogeneity in Computing Workshop, United States, 2017-05-29. 15 submissions, 7 papers accepted (single-blind reviews). 27 total authors and 21 total PC members. homepage: `http://hcw.eecs.wsu.edu/`

13. HiPC'17: International Conference on High Performance Computing, Data, and Analytics, India, 2017-12-18. 184 submissions, 41 papers accepted (single-blind reviews). 168 total authors and 102 total PC members. homepage: `http://hipc.org/`

14. HotCloud'17: Workshop in Hot Topics in Cloud Computing, United States, 2017-07-10. 58 submissions, 19 papers accepted (single-blind reviews). 64 total authors and 24 total PC members. homepage: `https://www.usenix.org/conference/hotcloud17`

15. HotI'17: Annual Symposium on High-Performance Interconnects, United States, 2017-08-28. 39 submissions, 13 papers accepted (single-blind reviews). 44 total authors and 23 total PC members. homepage: `http://www.hoti.org/hoti25/archives/`

16. HotOS'17: Workshop on Hot Topics in Operating Systems, Canada, 2017-05-07. 94 submissions, 29 papers accepted (single-blind reviews). 112 total authors and 9 total PC members. homepage: `https://www.sigops.org/hotos/hotos17/`

17. HotStorage'17: Workshop on Hot Topics in Storage and File Systems, United States, 2017-07-10. 58 submissions, 21 papers accepted (single-blind reviews). 94 total authors and 21 total PC members. homepage: `https://www.usenix.org/conference/hotstorage17`

18. HPCA'17: The IEEE Symposium on High Performance Computer Architecture, United States, 2017-02-04. 224 submissions, 50 papers accepted (single-blind reviews). 215 total authors and 46 total PC members. homepage: `http://hpca2017.org`

19. HPCC'17: International Conference on High Performance Computing and Communications, Thailand, 2017-12-18. 176 submissions, 77 papers accepted (single-blind reviews). 287 total authors and 75 total PC members. homepage: `http://hpcl.seas.gwu.edu/hpcc2017/`

20. HPDC'17: International Symposium on High Performance Parallel and Distributed Computing, United States, 2017-06-28. 100 submissions, 19 papers accepted (single-blind reviews). 76 total authors and 46 total PC members. homepage: `http://www.hpdc.org/2017/`

21. ICAC'17: International Conference on Autonomic Computing, United States, 2017-07-18. 73 submissions, 14 papers accepted (single-blind reviews). 46 total authors and 63 total PC members. homepage: `http://icac2017.ece.ohio-state.edu/`

22. ICDM'17: International Conference on Data Mining, United States, 2017-11-19. 778 submissions, 72 papers accepted (double-blind reviews). 269 total authors and 219 total PC members. homepage: `http://icdm2017.bigke.org/`

23. ICPE'17: ACM/SPEC International Conference on Performance Engineering, Italy, 2017-04-22. 83 submissions, 29 papers accepted (single-blind reviews). 102 total authors and 42 total PC members. homepage: `https://icpe2017.spec.org/`

24. ICPP'17: International Conference on Parallel Processing, United Kingdom, 2017-08-14. 210 submissions, 60 papers accepted (single-blind reviews). 234 total authors and 136 total PC members. homepage: `http://www.icpp-conf.org/2017/index.php`

25. IGSC'17: International Green and Sustainable Computing Conference, United States, 2017-10-23. 23 papers accepted (single-blind reviews). 83 total authors and 48 total PC members. homepage: `http://igsc.eecs.wsu.edu/`

26. IISWC'17: International Symposium on Workload Characterization, United States, 2017-10-02. 83 submissions, 31 papers accepted (double-blind reviews). 121 total authors and 27 total PC members. homepage: `http://www.iiswc.org/iiswc2017/index.html`

27. IMC'17: Internet Measurement Conference, United Kingdom, 2017-11-01. 179 submissions, 28 papers accepted (single-blind reviews). 124 total authors and 39 total PC members. homepage: `http://conferences.sigcomm.org/imc/2017/`

28. IPDPS'17: International Parallel and Distributed Processing Symposium, United States, 2017-05-29. 508 submissions, 116 papers accepted (single-blind reviews). 447 total authors and 286 total PC members. homepage: `http://www.ipdps.org/ipdps2017/`

29. ISC'17: ISC High Performance, Germany, 2017-06-18. 66 submissions, 22 papers accepted (double-blind reviews). 99 total authors and 55 total PC members. homepage: `http://isc-hpc.com/id-2017.html`

30. ISCA'17: International Symposium on Computer Architecture, Canada, 2017-06-24. 322 submissions, 54 papers accepted (double-blind reviews). 295 total authors and 70 total PC members. homepage: `http://isca17.ece.utoronto.ca/doku.php`

31. ISPASS'17: IEEE International Symposium on Performance Analysis of Systems and Software, United States, 2017-04-24. 81 submissions, 24 papers accepted (double-blind reviews). 98 total authors and 39 total PC members. homepage: `http://www.ispass.org/ispass2017/`

32. KDD'17: SIGKDD Conference on Knowledge Discovery and Data Mining, Canada, 2017-08-15. 747 submissions, 64 papers accepted (single-blind reviews). 237 total authors and 364 total PC members. homepage: `http://www.kdd.org/kdd2017/`

33. MASCOTS'17: International Symposium on the Modeling, Analysis, and Simulation of Computer and Telecommunication Systems, Canada, 2017-09-20. 84 submissions, 20 papers accepted (single-blind reviews). 75 total authors and 61 total PC members. homepage: `http://mascots2017.cs.ucalgary.ca/`

34. MICRO'17: International Symposium on Microarchitecture, United States, 2017-10-16. 327 submissions, 61 papers accepted (double-blind reviews). 306 total authors and 61 total PC members. homepage: `https://www.microarch.org/micro50/`

35. Middleware'17: The Annual Middleware Conference, United States, 2017-12-11. 77 submissions, 20 papers accepted (double-blind reviews). 91 total authors and 53 total PC members. homepage: `http://2017.middleware-conference.org/`

36. MobiCom'17: International Conference on Mobile Computing and Networking, United States, 2017-10-17. 186 submissions, 35 papers accepted (double-blind reviews). 164 total authors and 60 total PC members. homepage: `https://sigmobile.org/mobicom/2017/`

37. NDSS'17: The Network and Distributed System Security Symposium, United States, 2017-02-26. 423 submissions, 68 papers accepted (double-blind reviews). 327 total authors and 63 total PC members. homepage: `https://www.ndss-symposium.org/ndss2017/`

38. NSDI'17: USENIX Symposium on Networked Systems Design and Implementation, United States, 2017-03-27. 254 submissions, 42 papers accepted (double-blind reviews). 203 total authors and 42 total PC members. homepage: `https://www.usenix.org/conference/nsdi17/`

39. OOPSLA'17: International Conference on Object-Oriented Programming, Systems, Languages and Applications, Canada, 2017-10-25. 223 submissions, 66 papers accepted (double-blind reviews). 232 total authors and 30 total PC members. homepage: `https://2017.splashcon.org/track/splash-2017-OOPSLA`

40. PACT'17: International Conference on Parallel Architectures and Compilation Techniques, United States, 2017-09-11. 108 submissions, 25 papers accepted (double-blind reviews). 89 total authors and 33 total PC members. homepage: `https://parasol.tamu.edu/pact17/`

41. PLDI'17: SIGPLAN Conference on Programming Language Design and Implementation, Spain, 2017-06-18. 322 submissions, 47 papers accepted (double-blind reviews). 173 total authors and 56 total PC members. homepage: `http://pldi17.sigplan.org/home`

42. PODC'17: Symposium on Principles of Distributed Computing, United States, 2017-07-25. 154 submissions, 38 papers accepted (single-blind reviews). 101 total authors and 38 total PC members. homepage: `https://www.podc.org/podc2017/`

43. PODS'17: Symposium on Principles of Database Systems, United States, 2017-05-14. 101 submissions, 29 papers accepted (single-blind reviews). 91 total authors and 21 total PC members. homepage: `http://sigmod2017.org/pods-program/`

44. PPoPP'17: SIGPLAN Symposium on Principles and Practice of Parallel Programming, United States, 2017-02-04. 132 submissions, 29 papers accepted (double-blind reviews). 122 total authors and 44 total PC members. homepage: `http://ppopp17.sigplan.org/`

45. SC'17: The International Conference for High Performance Computing, Networking, Storage and Analysis, United States, 2017-11-13. 327 submissions, 61 papers accepted (double-blind reviews). 325 total authors and 213 total PC members. homepage: `http://sc17.supercomputing.org/`

46. SIGCOMM'17: SIGCOMM Conference, United States, 2017-08-21. 250 submissions, 36 papers accepted (double-blind reviews). 216 total authors and 50 total PC members. homepage: `http://conferences.sigcomm.org/sigcomm/2017/`

47. SIGIR'17: International Conference on Research and Development in Information Retrieval, Japan, 2017-08-07. 362 submissions, 78 papers accepted (single-blind reviews). 264 total authors and 283 total PC members. homepage: `http://sigir.org/sigir2017/`

48. SIGMETRICS'17: SIGMETRICS, United States, 2017-06-05. 203 submissions, 27 papers accepted (double-blind reviews). 101 total authors and 59 total PC members. homepage: `http://www.sigmetrics.org/sigmetrics2017`

49. SIGMOD'17: International Conference on Management of Data, United States, 2017-05-14. 489 submissions, 96 papers accepted (double-blind reviews). 335 total authors and 176 total PC members. homepage: `http://sigmod2017.org/`

50. SLE'17: International Conference on Software Language Engineering, Canada, 2017-10-23. 57 submissions, 24 papers accepted (single-blind reviews). 68 total authors and 28 total PC members. homepage: `http://www.sleconf.org/2017/`

51. SOCC'17: Symposium on Cloud Computing, United States, 2017-09-25. 45 papers accepted (single-blind reviews). 195 total authors and 52 total PC members. homepage: `https://acmsocc.github.io/2017/`

52. SOSP'17: Symposium on Operating Systems Principles, China, 2017-10-29. 232 submissions, 39 papers accepted (double-blind reviews). 217 total authors and 56 total PC members. homepage: `https://www.sigops.org/sosp/sosp17/`

53. SP'17: Security and Privacy, United States, 2017-05-22. 419 submissions, 60 papers accepted (double-blind reviews). 287 total authors and 62 total PC members. homepage: `https://www.ieee-security.org/TC/SP2017/index.html`

54. SPAA'17: Symposium on Parallelism in Alogirmths and Architectures, United States, 2017-07-24. 127 submissions, 31 papers accepted (single-blind reviews). 84 total authors and 40 total PC members. homepage: `http://spaa.acm.org/2017/index.html`

55. SYSTOR'17: International Systems and Storage Conference, Israel, 2017-05-22. 47 submissions, 16 papers accepted (single-blind reviews). 64 total authors and 33 total PC members. homepage: `https://www.systor.org/2017/`

56. VEE'17: International Conference on Virtual Execution Environments, China, 2017-04-09. 43 submissions, 18 papers accepted (double-blind reviews). 85 total authors and 19 total PC members. homepage: `http://conf.researchr.org/home/vee-2017`
